# Enhancing Solid-State Li-Ion Batteries with MOF–Polymer Composite Electrolytes—Effect Mechanisms and Interface Engineering

**DOI:** 10.3390/gels11120946

**Published:** 2025-11-25

**Authors:** Tao Chen, Nandarapu Purushotham Reddy, Man Li

**Affiliations:** 1 School of Materials Engineering, Changzhou Vocational Institute of Industry Technology, No.28 Mingxin Middle Road, Wujing District, Changzhou 213164, China; 2 Department of Electronic Engineering, Yeungnam University, Gyeongsan 38541, Gyeongbuk, Republic of Korea; npurush1992@gmail.com; 3 Department of Physics and Semiconductor Science, Gachon University, Seongnam-si 13120, Gyeonggi-do, Republic of Korea

**Keywords:** polymer composite electrolyte, metal-organic frameworks, solid-state battery, interfacial resistance, lithium-ion transport

## Abstract

Solid-state batteries (SSBs) are regarded as one of the most promising next-generation energy storage technologies due to their high energy density and improved safety. To achieve this goal, the development of solid-state electrolytes with high ionic conductivity and low interfacial resistance is essential. In recent years, composite polymer electrolytes (CPEs) have garnered extensive attention due to their ability to combine the intrinsic flexibility of polymers with the enhanced ionic conductivity and mechanical robustness provided by inorganic fillers. Metal–organic frameworks (MOFs), characterized by tunable pore structures, high surface areas, and excellent thermal and mechanical stability, are considered ideal fillers for constructing MOF–polymer composite electrolytes (MPCEs). This review summarizes the performance enhancement mechanisms of MPCEs and strategies for electrode–electrolyte interface stability. First, the primary preparation methods of MPCEs are introduced. Subsequently, the roles of MOFs in regulating ionic transport, suppressing dendrite growth, improving electrochemical stability, and optimizing the solid electrolyte interphase (SEI) layer are discussed. In addition, various interface engineering strategies are highlighted, including in situ polymerization of the polymer matrix, in situ growth of MOF fillers, integration of liquid plasticizers forming gel-like ionic conductor, and design of composite electrode to enhance interfacial compatibility and stability. Finally, the significant challenges and future research directions of MPCEs are outlined. This review provides valuable insights into the rational design of MPCEs and offers guidance for the development and practical application of high-performance SSBs.

## 1. Introduction

Among various electrolyte systems, liquid electrolytes (IEs) exhibit the highest ionic conductivity and excellent interfacial contact with electrodes [1,2]. They are widely used for most commercial lithium-ion batteries (LIBs). However, IEs’ inherent safety risks, low electrochemical stability, and susceptibility to dendrite growth have driven the search for alternative electrolyte systems [3,4]. In solid-state electrolytes (SSEs), inorganic ceramic electrolytes (ICEs) [5,6,7] (e.g., LATP, LLZO, LAGP) and composite materials generally exhibit higher ionic conductivity (σ) and superior lithium-dendrite suppression compared with solid polymer electrolytes (CPEs), owing to their enhanced mechanical strength and higher lithium-ion transference numbers (tLi^+^). Nevertheless, conventional polymer electrolytes [8,9] (e.g., PEO–LiTFSI, PVDF–LiTFSI) possess greater flexibility, better interfacial compatibility with electrodes, and excellent film-forming properties, making them easier to process and suitable for large-scale fabrication. The primary limitation of SPEs is their low ionic conductivity at room temperature (10^−6^–10^−5^ S cm^−1^), which arises from restricted ion mobility within the solid polymer matrix. To overcome these limitations, inorganic fillers such as Al_2_O_3_, TiO_2_, and LLZO have been incorporated into polymer matrices to form composite polymer electrolytes (CPEs) [10,11]. These fillers effectively enhance ionic conductivity and mechanical strength. Unfortunately, conventional inorganic–polymer CPEs still show limited improvement in electrochemical stability, particularly under high-voltage conditions.

Metal–organic frameworks (MOFs), owing to their unique structural and chemical features, have emerged as promising candidates for incorporation into CPEs to develop MOF-polymer composite electrolytes (MPCEs). The introduction of MOF fillers can disrupt the crystalline regions of polymers, thereby enhancing ion migration. Moreover, their crystalline architectures offer tunable pore sizes and geometries, which enable the construction of continuous ionic conduction networks with high ionic conductivity, thereby facilitating efficient ion transport [12]. In addition, the hybrid inorganic–organic nature of MOFs ensures good compatibility with polymer matrices. At the same time, their incorporation as reinforcing fillers can effectively broaden the electrochemical stability window of composite electrolytes. Finally, Lewis acid–base interactions within MOF/polymer composites further improve their overall electrochemical stability [13,14,15]. Compared to SPEs and ICEs, the fabrication of MPCEs is more complex, as it requires the effective integration of MOFs and polymer matrices through multistep processes, such as solution casting, electrospinning, or in situ polymerization. Nevertheless, MPCEs combine the potential advantages of both MOFs and polymers, exhibiting a series of promising properties, including high ionic conductivity, excellent mechanical flexibility, and enhanced electrochemical working window and chemical stability. As a result, the integration of MOFs with polymer electrolytes has emerged as an auspicious and innovative research direction, becoming one of the most attractive topics in the field of SSE development, despite the existing challenges. Despite the rapid progress achieved in recent years, MPCEs still suffer from severe limitations arising from large interfacial resistance and unstable electrode–electrolyte interfaces, which lead to reduced energy density and long-term cycling stability [16,17]. Typically, the main interfacial issues include: (i) poor electrolyte–electrode contact resulting in high interfacial resistance; (ii) structural stress and interfacial degradation caused by periodic volume changes of the electrodes and electrolytes; (iii) parasitic reactions such as polymer and salt decomposition that deteriorate the chemical stability at the interface; and (iv) compositional inhomogeneity within the electrolyte that causes poor interfacial compatibility [16]. These factors collectively result in non-uniform ion transport and severe local polarization, triggering dendrite growth and capacity fading during cycling. Therefore, constructing a tightly bonded and stable interface between MPCEs and electrodes is a critical challenge that must be overcome to achieve SSBs. Many studies have focused on addressing these issues through synergistic strategies, including structural optimization, chemical modification of the electrolytes, and interface engineering of electrode materials [18,19,20]. This review aims to provide a timely and distinctive analysis of recent advances in MPCEs. While other reviews have covered MOF-based electrolytes, our work sets itself apart by providing a structured and critical comparison of four key interface engineering strategies: in situ polymerization, in situ MOF growth, gel-like ionic conductors, and composite cathode design. We place a strong emphasis on practical performance metrics, such as room-temperature ionic conductivity, Li^+^ transference number, critical current density, and cycling stability under relevant conditions, to evaluate the true progress towards viable SSBs. Here, we summarize recent advances in the fabrication of MPCEs and highlight the enhancement mechanisms of MOFs within polymer matrices. In particular, the interfacial engineering strategies for MPCEs to improve interfacial compatibility and stability between electrolyte and electrodes are emphasized, including optimization of MPCEs synthesis routes, formation of gel-like ionic conductor by introducing liquid plasticizers to improve interfacial adaptability, and modification of composite cathode design to reduce interfacial resistance, all of which have led to significant progress in the field.

## 2. MOF-Polymer Composite Electrolytes (MPCEs)

### 2.1. Design and Synthesis Strategies of MPCEs

**Figure 1:**
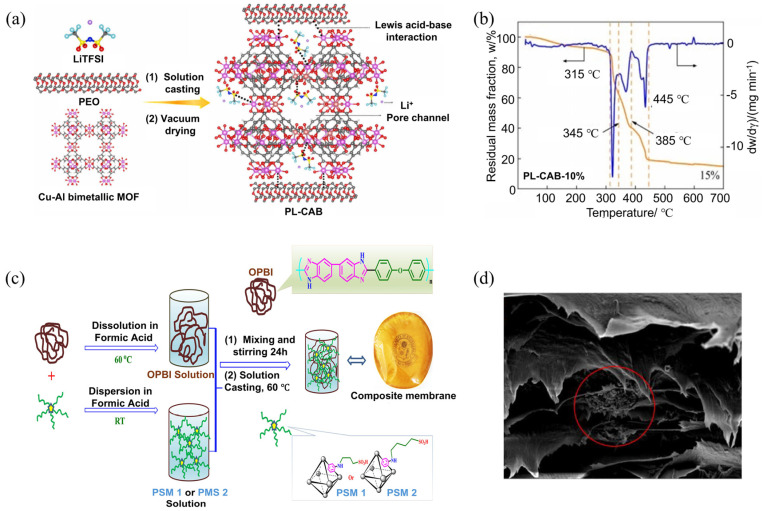
Solution casting method for composite electrolyte preparation. (**a**) Schematic diagram of synthesis and (**b**) thermogravimetric curves of PL-CAB electrolyte [24]. (**c**) Preparations of PSM 1/PSM2-OPBI composite Membrane. (**d**) SEM cross-sectional image of composite film made via solution casting method (red circle shows the MOF fillers within the film) [25].

MPCEs are hybrid systems constructed by incorporating MOFs with high porosity, large surface area, and tunable chemical environments into polymer matrices. The polymer matrix primarily provides mechanical support and serves as an ion-transport medium, while MOFs act as functional fillers or structural skeletons that regulate ion conduction and interfacial stability at the microscopic level. Among various fabrication strategies, physical blending is the most commonly employed and straightforward approach. This method enables the homogeneous dispersion of MOF particles within the polymer matrix through simple physical processes without damaging the intrinsic crystalline framework of MOFs, thereby combining the flexibility of polymers with the rigidity of inorganic fillers [21]. Three major types of physical blending methods are summarized as follows:

*Solution Casting*: The solution casting method is a simple and widely used physical blending strategy for preparing CPE membranes. This technique offers several advantages, including a straightforward process, easy control of component ratios, and suitability for large-scale production [22,23]. Consequently, it is generally regarded as a standard experimental route for fabricating polymer composite electrolytes. In the preparation of MPCEs, the polymer is typically dissolved in a polar solvent (such as acetonitrile, DMF, or NMP), followed by the sequential addition of a lithium salt and MOF fillers. After achieving a homogeneous dispersion through mechanical stirring or ultrasonic treatment, the resulting solution is cast into a mold and the solvent is evaporated under vacuum at elevated temperature, yielding a dense composite solid electrolyte membrane. For instance, Song et al. [24] combined Cu–Al bimetallic MOFs (CAB) with polyethylene oxide (PEO) and lithium salts to prepare a composite electrolyte (PL-CAB) via the solution casting method (Figure 1a). The CAB MOF possesses abundant and highly Lewis-acidic metal sites, which can interact with the oxygen atoms in both the PEO chains and the TFSI^−^ anions. Such interactions disrupt the crystalline structure of PEO, reduce its crystallinity, and consequently enhance the ionic conductivity. Meanwhile, this coordination effect effectively restricts anion migration and improves Li⁺ transport capability. In addition, compared with the pristine PEO-LiTFSI (PL) electrolyte, the incorporation of MOF fillers markedly enhances the thermal stability of the composite electrolyte (Figure 1b). Similarly, Mukhopadhyay et al. [25] mixed post-synthetically modified (PSM) MOFs (named as PSM 1 and PSM) with ether-type polybenzimidazole (OPBI) polymer and subsequently applied the same solution casting method to produce the composite film (Figure 1c) [8]. The SEM image in Figure 1d shows that the MOF fillers are scattered into the cross-section of composite membranes, which can significantly improve conductivity as well as mechanical strength.

**Figure 2:**
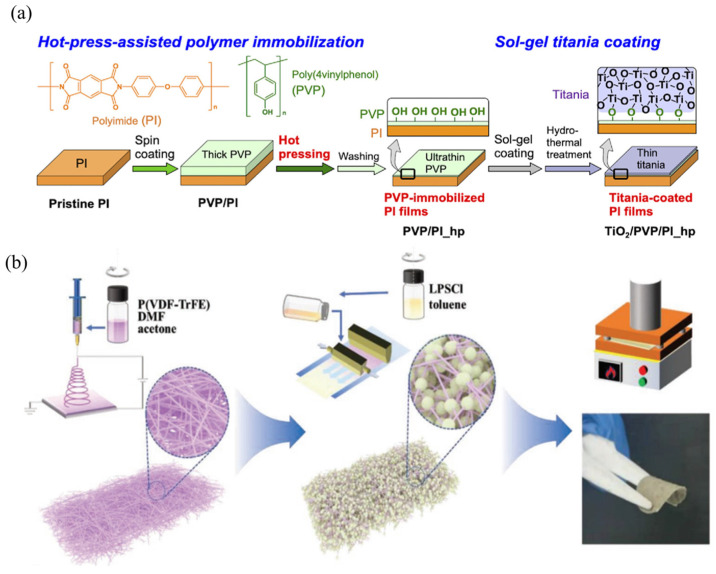
Hot pressing method for composite electrolyte. (**a**) The polymer surface modification using the hot pressing method [27]. (**b**) The fabrication process of the LPSCl@P(VDF-TrFE) electrolyte membrane via an electrospinning-infiltration hot-pressing method [28].

*Hot Pressing:* The hot-pressing method is another commonly employed physical compression technique for fabricating composite solid electrolytes. Its fundamental principle involves uniformly mixing a polymer, lithium salt, and inorganic filler, followed by pressing the mixture in a mold under elevated temperature (approximately 80–150 °C) and pressure (about 5–10 MPa). This solvent-free process eliminates the issue of residual solvents and enables the fabrication of composite electrolytes with high mechanical strength and enhanced interfacial contact [26]. Gerbaldi and co-workers dispersed an appropriate amount of Al-BTC MOF into a PEO–LiTFSI matrix and used an Ultra-Turrax^®^ high-speed mechanical stirrer for 3 min to ensure complete dispersion of the MOF filler [15]. The homogeneous mixture was then hot-pressed in an aluminum mold at 80–100 °C to obtain uniform and dense composite electrolyte films with an average thickness of 30 ± 10 μm. Differential scanning calorimetry (DSC) analysis revealed that the incorporation of MOFs increased the melting temperature of the electrolyte from 59 °C to 66 °C, while the glass-transition temperature (Tg) rose from −54 °C to −48 °C, indicating an enhancement in mechanical rigidity. Thermogravimetric analysis (TGA) further showed that no significant weight loss occurred before the onset of irreversible decomposition (~345 °C), confirming the excellent thermal stability of the composite electrolyte films, which can safely operate at temperatures slightly above those typical for lithium batteries. In addition, Hashizume’s work showed that the hot pressing method can assist inert polymer surface modification (Figure 2a), leading to free damage to the polymer substrate [27]. Furthermore, the hot-pressing process has also been widely applied to other CPEs containing inorganic fillers. For instance, Liu et al. [28] employed a combined electrospinning–infiltration–hot-pressing method in which sulfide particles were infiltrated into a porous polymer framework, followed by hot pressing to fabricate a composite electrolyte consisting of argyrodite-type Li_6_PS_5_Cl and a polar PVDF-TrFE backbone (Figure 2b). The resulting membrane exhibited an ionic conductivity of approximately 1.2 mS cm^−1^ at room temperature, and the assembled LPSCl@P(VDF-TrFE) solid-state battery retained 71% of its capacity after 20,000 cycles at a current density of 1.0 mA cm^−2^, demonstrating outstanding cycling stability and structural integrity.

**Figure 3:**
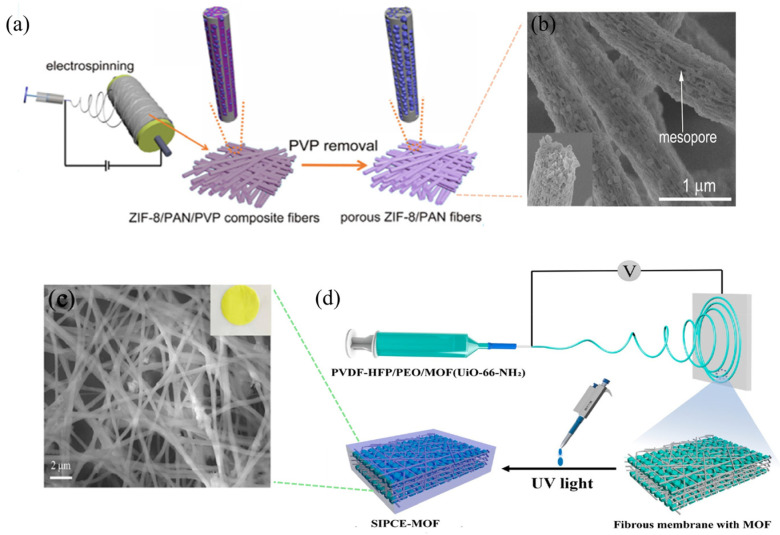
Electrospinning method for composite electrolytes. (**a**) Fabrication procedure and (**b**) SEM image of porous ZIF/PAN fiber [30]. (**c**) Schematic illustration of design and preparation of SIPCE-MOF, (**d**) SEM image and digital photo of fabricated SIPCE-MOF film [12].

*Electrospinning:* Electrospinning is an electrostatic field–driven technique for fabricating nanofiber membranes characterized by high specific surface area, ultrathin thickness, remarkable porosity, and excellent flexibility [29]. In the fabrication of MPCEs, MOF powders or precursors are uniformly dispersed into a polymer–lithium-salt solution. A strong electric field is generated under a high voltage (typically 10–25 kV) between the needle and the collector, stretching the mixed solution into continuous nano- to microscale fibers that deposit on the collector to form a nonwoven porous membrane. This process constructs a three-dimensional (3D) network with high surface area, interconnected ion pathways, and shortened diffusion distances, thereby enhancing ionic conductivity, reducing interfacial resistance, and improving overall mechanical flexibility. For instance, Zhao et al. [30] prepared a porous ZIF-8/PAN composite nanofiber membrane by dispersing polyacrylonitrile (PAN) and ZIF-8 in DMF with polyvinylpyrrolidone (PVP) as a sacrificial pore-forming agent, followed by electrospinning under 18 kV at a feed rate of 1 mL h^−1^ (Figure 3a,b). In Chen’s work [12], a DMF suspension of UiO-66-NH_2_ was mixed with a DMF solution of Poly(vinylidene fluoride-co-hexafluoropropylene) (PVDF-HFP)/PEO and stirred for 12 h to obtain a homogeneous electrospinning solution. The solution was then electrospun at room temperature under 20 kV with a feed rate of 0.1 cm min^−1^, yielding a uniform MOF/polymer composite fibrous membrane. Subsequently, an in situ photo-initiated polymerization was carried out to graft single-ion polymer monomers onto the fiber surface, forming a novel single-ion polymer composite electrolyte (SIPCE) (Figure 3c,d). Compared with conventional polymer electrolytes, SIPEs exhibit higher lithium-ion transference numbers, improved interfacial compatibility with lithium-metal anodes, and effective suppression of lithium dendrite growth.

*Practical advantages and limitations:* In the context of solution casting, hot-pressing, and electrospinning, these techniques are commonly employed for fabricating MPCEs, resulting in various performance improvements. However, it is essential to consider the practical limitations of each method. Solution casting offers a scalable and straightforward approach; however, it may leave residual solvent and generally produces limited MOF dispersion unless extended sonication or surface functionalization is used, which increases complexity for scale-up [22,25]. Hot-pressing is a solvent-free technique that yields dense films with excellent interfacial adhesion. Nevertheless, high temperatures and pressures can cause polymer chain reorganization, affect the crystallinity of metal-organic frameworks, and hinder the addition of temperature-sensitive fillers [26,27]. Electrospinning creates fibrous networks with a high surface area, which enhances wettability and reduces the length of ion pathways. Still, ensuring consistent loading of MOFs and effectively removing binders, mainly when sacrificial porogens are used, remains challenging. Furthermore, fiber thinning could potentially weaken the mechanical strength when subjected to stacking pressure. Comprehensive investigations measuring dispersion, residual solvent, and mechanical stability under realistic cell pressures are essential to support the transition to practical cells.

### 2.2. Effect Mechanisms of MOF in MPCEs

#### 2.2.1. Ion Transport Regulation

The ionic conductivity (σ) and lithium ion transference number (tLi^+^) are core variables that describe solid-state electrolytes’ ion transport capability, directly determining the charge-discharge efficiency and cycling stability of SSBs [31,32]. Ionic conductivity is an indicator of ionic species’ mobility within the electrolyte—Li^+^, anions, and any other impurity ions. High ionic conductivity implies higher migration rates of the ions in the electrolyte, resulting in reduced internal resistance and increased rate capability. In contrast, an ion transference number quantifies the fraction of an ion—here, predominantly Li^+^—which contributed to total ionic conduction. In most electrolytic systems, the contribution to charge transport is from both Li^+^ and an anion (e.g., TFSI^−^). However, excessive anion transport leads to concentration polarization, wherein differential accumulation of Li^+^ near the electrode surface increases the local current density and promotes lithium dendrite formation. If the Li^+^ transference number (tLi^+^) approaches 1, there is predominant transport by Li^+^, while anion migration is weakly suppressed, significantly reducing concentration polarization and dendrite growth. Compared to traditional inert inorganic fillers like Al_2_O_3_ and SiO_2_, MOFs afford remarkable opportunities to control ion transport behavior due to their highly tunable structure and chemical functionality. Connections between MOF structure and ion transport are crucial. The trends in reported MPCEs (Table 1) show that three MOF features most significantly influence σ and tLi⁺: (i) Pore size and connectivity—continuous sub-nanometer to nanometer channels create “preferential” pathways for Li⁺, reducing tortuosity; (ii) Surface chemistry (open metal sites, polar groups)—Lewis acidic metal sites or polar linkers coordinate anions like TFSI^−^, increasing the fraction of free Li⁺ and enhancing tLi⁺, as supported by solid-state NMR and molecular dynamics studies [13,33]; and (iii) Morphology/aspect ratio—ultrathin nanosheets or well-dispersed nanocrystals more effectively disrupt polymer crystallinity and lower segmental hopping distances. Table 1 highlights key quantitative examples, with σ values ranging from 10^−4^ to 10^−3^ S cm^−1^ and tLi⁺ values of around 0.4 to 0.9, depending on the specific MOF and polymer [31].

Integrated conceptual framework. We propose an easy-to-follow mapping for practical design: (A) Metal-organic frameworks with many open metal sites and hydrophilic or polar linkers facilitate anion immobilization, which enhances lithium ion transport; (B) frameworks with hierarchical interconnected pore networks that enable efficient polymer infiltration form continuous ionic channels, improving conductivity; (C) large two-dimensional nanosheets or well-dispersed nanoparticles help significantly reduce polymer crystallinity, boosting both conductivity and lithium ion transport, though mechanical reinforcement is needed to prevent dendrite penetration. This framework guides the selection of specific experiments (like systematic pore-size series or controlled linker functionalization) to turn heuristics into predictive models. The ordered porous structure provides continuous pathways for Li^+^ conduction. At the same time, the abundance of surface-active sites promotes the dissociation of the lithium salt, thereby increasing the concentration of free Li^+^ ions and enhancing ion mobility, which significantly improves the ionic conductivity of the electrolyte. Simultaneously, the open metal sites or polar ligands within the MOFs can selectively adsorb anions, such as TFSI^−^ and FSI^−^, contributing to the high tLi^+^. For instance, incorporating UIO-66-NaSO_3_-type MOFs into a polyethylene oxide (PEO) matrix markedly enhanced the ionic conductivity, achieving 1.67 × 10^−3^ S·cm^−1^ at 60 °C, which is substantially higher than that of pristine PEO electrolytes [34]. Similarly, a PEO-based composite electrolyte modified with a Cu–Al bimetallic MOF (CAB) exhibited a significantly increased Li⁺ transference number of 0.459 at 60 °C, far exceeding that of pure PEO, demonstrating the synergistic role of MOFs in simultaneously improving σ and tLi⁺ [24]. The enhanced ionic conductivities and tLi^+^ parameters for different reported MOF-filled polymer electrolytes are summarized in Table 1. All the ionic conductivities were measured via the AC impedance curves, and all the tLi^+^ values were tested using the constant voltage polarization method in the table.

**Table 1:** The ionic conductivities and tLi^+^ parameters of reported polymer composite electrolytes incorporating MOFs.

MPCEs	Ionic Conductivity (σ) (S cm^−1^)	Lithium Ion Transference Number (tLi^+^)	Ref.
PVDF-HFP@ZIF-8@PVPG	0.55 × 10^−3^ (22 °C)	0.87	[35]
PVDF-HFP-Cu-MOF-74	7.9 × 10^−4^ (25 °C)	0.69	[36]
PVDF-HFP-Ni,Go-MOF	0.68 × 10^−3^ (25 °C)	0.49	[37]
ZIF-8/polyether (F127)	0.74 × 10^−4^ (30 °C)	0.58	[38]
PVDF-HFP-UIO-66	6.9 × 10^−4^ (25 °C)	0.59	[39]
PVDF-HFP-UIO-66	3.37 × 10^−4^ (25 °C)	0.90	[40]
PVDF-HFP-ZIF-8	0.46× 10^−3^ (25 °C)	0.74	[41]
PAN-HKUST-1	2.40 × 10^−3^ (25 °C)	0.698	[42]
PDMA-MOF-808	10^–4^ (25 °C)	0.77	[33]
PVDF-HFP-UiO-66	5.55 × 10^–4^ (25 °C)	0.52	[43]
PolyDOL (PDOL)-fluorinated-UIO66	3.96 × 10^–4^ (25 °C)	0.65	[44]
PEO-UIO-66@67	9.2 × 10^−4^ (25 °C)	0.74	[45]

#### 2.2.2. Dendrite Suppression

**Figure 4:**
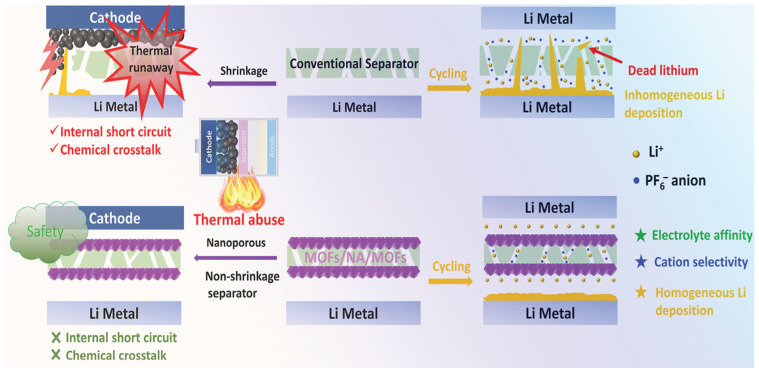
Schematic diagram for the working principle of MOFs suppressing lithium dendrites and improving the battery safety [48].

Lithium dendrites are tree-like metallic lithium formations on the surface of lithium-metal anodes due to uneven deposition of Li⁺ during repeated charge–discharge cycles. These are one of the most serious obstacles to the performance and safety of lithium-metal batteries, causing internal short circuits, capacity fading, and low Coulombic efficiency (CE). Apart from that, the random and uncontrolled growth of dendrites causes a significant volume change within the lithium-metal anode, compromising the integrity of the anode/electrolyte interface. Interfacial voids, hence created, prevent the electrode from being wetted adequately by the electrolyte, causing increased capacity degradation issues and compromising long-term cycling stability [46,47]. In terms of effective strategies for dendrite suppression, they include achieving uniform Li⁺ deposition, increasing the mechanical strength of electrolytes, and minimizing parasitic reactions at the anode interface. In this respect, the MOF possesses distinct advantages: A well-defined porous channel can regulate the Li⁺ flux and build up a uniform electric-field distribution that supports homogeneous Li nucleation and deposition. Additionally, when MOFs are used as fillers in CPEs, significant mechanical strengthening occurs, and physical barriers are provided against the growth and penetration of dendrites. Liu and co-workers designed a sandwich-structured MOF/PEN@PDA/MOF multifunctional separator, where ordered anion-philic MOF layers were grafted onto both sides of a porous poly(arylene ether nitrile) (PEN) membrane pre-modified with polydopamine (PDA) [48]. Benefiting from the anion-affinitive properties and uniform microporous morphology of the MOF layers, density functional theory (DFT) calculations confirmed that anion migration was effectively restricted, enabling efficient and homogeneous Li⁺ diffusion. Consequently, the tLi⁺ increased markedly from 0.22 to 0.81. Furthermore, the excellent electrolyte affinity and absorption capability of MOFs helped maintain a uniform Li⁺ flux distribution throughout cycling. Electrochemical evaluations revealed that the MOF layer served as a “Li⁺ guider”, effectively homogenizing the internal electric field, suppressing random anion migration, and prolonging the nucleation induction period of dendrite formation (Figure 4). As a result, the growth of lithium dendrites was effectively inhibited, enabling highly stable Li plating/stripping performance and improved cycling durability with a lifetime exceeding 500 h.

Constraints and Ongoing Issues in Dendrite Suppression. Although MOF layers and MOF-filled CPEs serve as practical Li⁺ “guiders” and mechanical barriers, there are still several practical challenges to address. (i) Mechanical fragility: numerous MOF crystals exhibit brittleness and limited compliance; when subjected to stack pressure or during repeated volume fluctuations of electrodes, they may fracture, compromising their intended guidance function. (ii) Chemical stability concerning lithium: specific MOFs (notably those featuring redox-active or Lewis-basic linkers) may interact with Li metal or its decomposition products, thereby affecting interfacial chemistry. (iii) Interfacial voids at elevated current densities: suboptimal wetting, microscopic detachment, and void formation during rapid stripping/plating can lead to the emergence of local hotspots that facilitate dendrite nucleation. To tackle these challenges, it is essential to employ a combination of mechanical techniques, such as nanoindentation and in situ pressure tests, alongside chemical analyses like XPS and ToF-SIMS following cycling, as well as operando imaging studies conducted under pertinent current densities. These considerations moderate expectations for swift application and underscore the necessity for focused experiments to demonstrate reliability [3,31].

#### 2.2.3. Electrochemical Stability Window

During high-voltage charging, side reactions often occur at the interface between the cathode and the SPE. This instability arises because transition metal ions (e.g., Ni^2^⁺, Co^3^⁺, Mn^4^⁺) released from the cathode or conductive additives such as carbon can trigger and catalyze the oxidative decomposition of the polymer matrix, thereby reducing the oxidative stability of the SPE and leading to interfacial degradation and capacity fading [49]. CPEs incorporating inorganic fillers exhibit improved high-voltage stability compared with pristine polymer electrolytes. This enhancement is primarily attributed to the presence of inorganic fillers, such as MOFs, which introduce spatial confinement and surface interactions with lithium ions. These effects modulate the coordination between Li⁺ and the oxygen atoms in the polymer chains, making the polymer backbone more resistant to oxidation and increasing the decomposition voltage of the CPE [31]. Furthermore, synergistic modification of both the polymer matrix and lithium salts can further enhance the high-voltage stability of the electrolyte. According to molecular orbital theory, a polymer with a lower highest occupied molecular orbital (HOMO) energy level exhibits greater voltage stability, particularly in terms of oxidative resistance under high-voltage conditions. PVDF-HFP possesses a low HOMO level and a high dielectric constant, demonstrating good stability under high-voltage operation, thereby becoming one of the most extensively studied polymer electrolytes [50,51]. It is worth noting that the fundamental interaction mechanisms between fillers and polymer matrices remain under debate and require further experimental and theoretical investigation. Currently, the enhanced electrochemical stability of CPEs is widely attributed to various interfacial interactions, including Lewis acid–base interactions between filler functional groups and polymer lone-pair electrons, hydrogen bonding, and dipole–dipole interactions [52]. These combined effects modify the polymer’s electronic environment (e.g., HOMO energy level), thereby broadening the electrochemical stability window. In particular, MOF fillers, owing to their high surface area and tunable metal nodes and organic linkers, provide abundant Lewis acidic sites that strongly interact with the polymer matrix, effectively enhancing the electrochemical stability and expanding the electrochemical window of the composite electrolyte [53,54].

**Figure 5:**
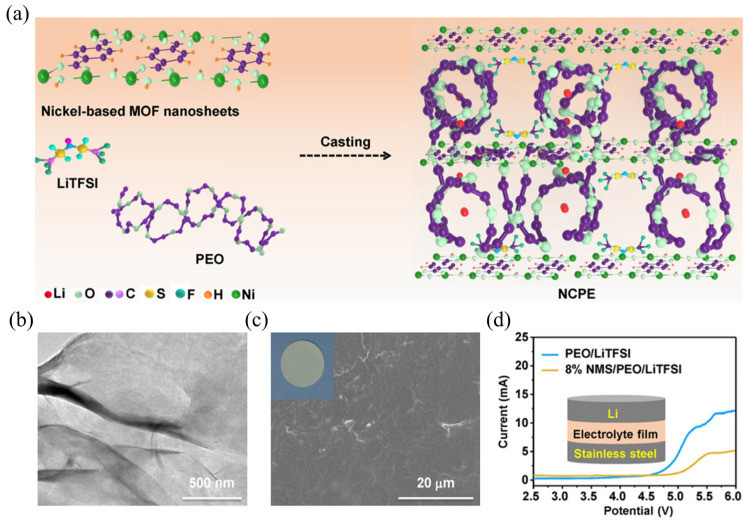
(**a**) Schematic illustration of CPE with NMS in PEO/LiTFSI electrolyte. (**b**) TEM image of NMS. (**c**) Surface SEM image and digital photography of NCPE film. (**d**) LSV curves of the cells using CPE and NCPE at 25 °C [55].

Han et al. [55] synthesized nickel-based ultrathin MOF nanosheets (named NMSs) with excellent structural stability using a simple ultrasonic-assisted method and incorporated them into a PEO-based electrolyte (Figure 5a–c). The high aspect ratio of the NMSs effectively suppresses the regular folding and ordered packing of PEO chains, thereby reducing the crystallinity of PEO. The ultrathin nanosheet architecture (Figure 5b) also provides a shorter and more continuous ion-transport pathway at the CPE/NMS interface. Benefiting from these structural advantages, the ionic conductivity and lithium-ion transference number (σ = 1.66 × 10^−5^ S cm^−1^ at 25 °C; tLi^+^ = 0.378)” of the NMS-doped composite polymer electrolyte (NCPE) were significantly enhanced. Furthermore, the NMS consists of a coordination framework in which central Ni^2^⁺ ions are connected by BDC^2−^ (1,4-benzenedicarboxylate) organic linkers, serving as crosslinking centers for PEO and forming a stable electrolyte network. Equally important, the ultrathin NMS exposes abundant unsaturated Ni sites on its surface, providing Lewis acidic centers that suppress the diffusion of TFSI^−^ anions and thereby promote Li⁺ transport. In addition, the porous structure of the NMS can effectively remove interfacial impurities such as residual H_2_O, enabling the NCPE to exhibit an expanded electrochemical stability window of 4.9 V at 25 °C (Figure 5d) and superior electrochemical performance.

#### 2.2.4. Optimize Solid Electrolyte Interphase (SEI) Layer

**Figure 6:**
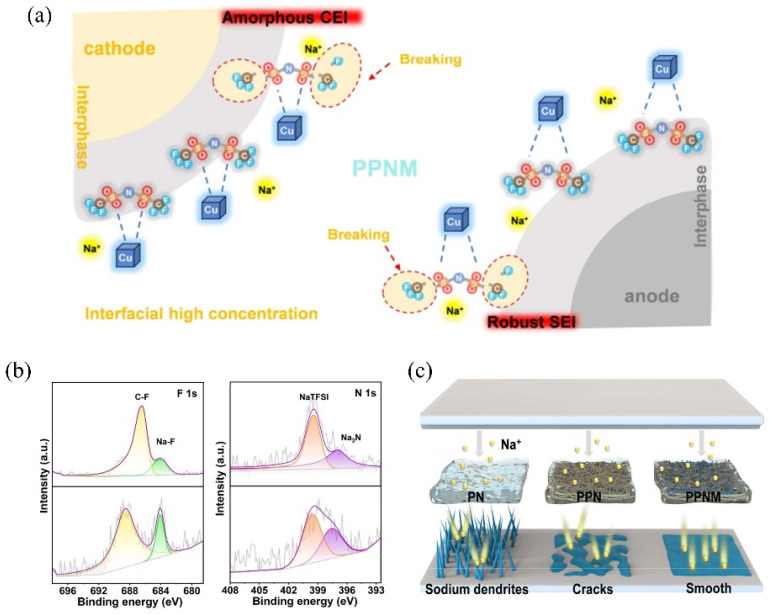
(**a**) Schematic illustration of interphase regulation for electrodes. (**b**) F 1s and N 1s XPS spectra of cycled Na anode in the symmetric cell with PPN and PPNM electrolytes. (**c**) Na ion deposition performance in the SSNBs with PN, PPN, and PPNM electrolytes [58].

PEO, one of the most widely used polymer electrolyte matrices for solid-state lithium metal batteries (SLMB), exhibits good chemical compatibility with lithium metal, forming a stable and ionically conductive solid electrolyte interphase (SEI) layer on the anode surface [8,56]. However, when PEO is employed in solid-state sodium-metal batteries (SSMBs) to develop the SPE, the highly reactive sodium metal tends to directly reduce and decompose the PEO chains [17,57]. This reaction will generate an unstable SEI composed of complex components (such as Na_2_O, Na_2_CO_3_, and ROCO_2_Na). Therefore, enhancing ionic conductivity and constructing a stable SEI remain significant challenges for PEO-based SPEs in SSNMs. Tian et al. [58] reported a strategy using the open metal sites (OMS) within Cu–MOFs to immobilize and promote the decomposition of TFSI^−^ anions, thereby optimizing the formation of a robust SEI layer and effectively suppressing the growth of sodium dendrites in SSMB (Figure 6a–c). An MPCE was successfully fabricated through electrospinning and solution casting, in which 3D interconnected Cu–MOFs were integrated onto polyacrylonitrile (PAN) fibers, combined with sodium bis(trifluoromethanesulfonyl)imide (NaTFSI) salt and PEO, forming the PPNM membrane. The strong anchoring effect of Cu–MOFs on TFSI^−^ facilitated anion decomposition at the electrode–electrolyte interface, leading to the formation of NaF- and NaN-rich cathode electrolyte interphase (CEI) and SEI layers, as evidenced by F1S, N1S XPS spectra in the Figure 6b. This mechanism, where MOF open metal sites immobilize anions and promote the formation of a beneficial SEI, is further supported by spectroscopic and electrochemical analysis [58]. Compared to pure PEO-based SPE (named as PN) and PEO/PAN-based SPE, the synergistic dual-interface regulation from PPNM-based MPCE significantly suppressed sodium dendrite growth, polymer degradation, and transition-metal dissolution (Figure 6a–c). The symmetric cell using the PPNM MPCE exhibited outstanding interfacial stability, operating for nearly 1000 h at 0.1 mA cm^−2^ with a capacity of 0.1 mAh cm^−2^. These results confirm that the MOF fillers in the polymer matrix promote interfacial stability.

## 3. Interface Engineering Using MPCEs

The interfacial stability between CPEs and electrodes is a key criterion for evaluating the effectiveness of CPEs and has a significant impact on the operational performance of SSBs [59,60,61,62]. A stable CPE/electrode interface ensures the reversible insertion and extraction of Li⁺, effectively minimizing interfacial side reactions and nonuniform Li⁺ deposition. This results in a markedly reduced internal resistance of SSBs and efficient suppression of excessive lithium dendrite growth, thereby extending the cycling lifetime of SSBs. Therefore, enhancing the interfacial stability of CPEs with electrodes remains a crucial research focus in the design and fabrication of SSBs. Although SPEs exhibit better interfacial compatibility with electrodes than other ICEs, some challenges still exist, making them insufficient for practical applications, including side reactions and uneven lithium deposition [63,64,65]. Incorporating MOFs as fillers into the polymer matrix can effectively enhance the interfacial compatibility and chemical stability of SPEs, significantly mitigating side reactions [14,66,67]. Moreover, MOFs with continuous and homogeneous Li⁺ transport channels can facilitate uniform lithium deposition and further enhance interfacial stability [68,69]. To facilitate comparison among the various interface engineering methods outlined below, Table 2 summarizes the primary mechanism, practical usability, implementation complexity, typical strengths and limitations, and representative performance ranges (ionic conductivity σ and tLi⁺ where available). This comparison emphasizes trade-offs—for example, in situ techniques offer better contact but are synthetically more complex; gel-like conductors improve wettability but may reduce mechanical robustness—and highlights areas where further knowledge is needed.

### 3.1. Interfacial Challenges in SSBs

#### 3.1.1. Fundamental Challenges for the Cathode–CPE Interface

In general, the large contact area between CPE and the cathode leads to poor oxidative stability of the CPE [73,74]. In SSBs, the CPE usually contacts cathode particles closely to improve ionic conductivity. This increases the actual surface area exposed to high potentials, typically above 4 V, which makes the polymer backbone more prone to oxidative breakdown. Additionally, the unsaturated coordination of transition-metal ions like Ni, Co, Mn, and Fe, along with defect sites in conductive carbon, such as graphitic edges and oxygen-containing functional groups, promotes the formation of radical or cationic intermediates. This process speeds up polymer oxidation. Under high-voltage conditions, polymers like PEO, acrylates, carbonate side chains, and ether linkages can create cationic radicals or carbocations. The following β-scission or hydrogen-transfer reactions weaken or activate the C–H bonds. The hydrogen atoms that are removed are captured by anions from the electrolyte salts, such as TFSI^−^, FSI^−^, BF_4_^−^, and DFOB^−^. This leads to the formation of acidic substances. These acids worsen interfacial side reactions at the cathode–CPE interface by corroding transition-metal oxide surfaces. This promotes transition-metal dissolution and migration, and further catalyzes polymer chain breaking or crosslinking. The result is the creation of viscous and ion-blocking degradation layers that harm electrochemical performance [16]. In addition, during the charging process, volume changes from lattice parameter shifts in layered or Ni-rich cathodes and phosphate-type materials cause microcracks and pore development within cathode particles and binders. This leads to poor interfacial compatibility with the CPE. On a microscopic level, nanoscale and microscale detachment zones appear, increasing local current density and creating reaction hotspots. Such incompatibility at the interface causes significant polarization and inevitably results in a space-charge layer (SCL). The uneven charge distribution leads to local stoichiometric changes and structural distortion, further raising the internal resistance of the battery [11,75]. Therefore, it is crucial to create various strategies to optimize both CPE and cathode materials, along with fabrication processes, to build a chemically and mechanically stable interface.

#### 3.1.2. Basic Challenges for the Li Metal Anode–CPE Interface

Lithium metal, with its exceptionally high theoretical specific capacity (3860 mAh g^−1^) and the lowest electrochemical potential (−3.04 V vs. SHE), is regarded as one of the most promising anode materials for next-generation batteries [76,77,78]. The use of lithium metal can significantly enhance the energy density of solid-state batteries (SSBs); however, interfacial challenges between lithium metal and the CPE remain unresolved. Although the CPE provides mechanical support and ionic conduction pathways, it is not fully compatible with lithium in terms of chemical and mechanical stability, exhibiting poor interfacial wettability, uneven ion transport, and insufficient chemical robustness. During cycling, the periodic expansion and contraction of lithium metal further aggravate the already weak mechanical contact with the CPE [79]. In addition, the instability of the electrolyte at low reduction potentials during lithium stripping induces undesirable side reactions. The polymer backbone of the CPE (e.g., ether, ester, and carbonate groups) and the anions of lithium salts (such as FSI^−^ and TFSI^−^) are prone to reduction, forming inorganic and organic species. These decomposition products are typically brittle and ionically insulating, preventing the formation of a stable solid–SEI [80,81]. The reaction products further corrode the lithium surface, generate “dead lithium,” and reduce the Coulombic efficiency. The resulting heterogeneous and ion-blocking interface causes poor interfacial contact and increased internal resistance. Finally, all these factors lead to charge redistribution at the Li–electrolyte interface, promoting the growth of lithium dendrites and the degradation of SSB performance [82,83].

### 3.2. Interfacial Engineering Strategies for MPCEs

#### 3.2.1. In Situ Polymerization

**Figure 7:**
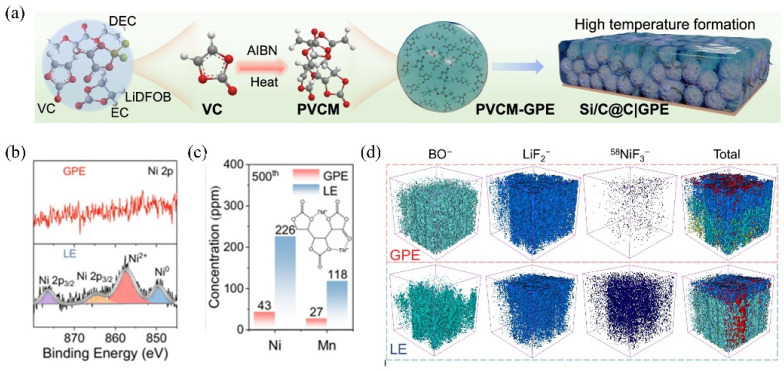
(**a**) In situ polymerization process for the PVCM-GPE preparation. (**b**) Ni 2p XPS spectra, (**c**) ICP-MS analysis of the cycled anode obtained from the full batteries PVCM-GPE and LE after 500 cycles. (**d**) TOF-SIMS 3D plots for BO^−^, LiF_2_^−^, ^58^NiF^3−^, and total construction [18].

In situ polymerization refers to the direct polymerization of monomers or polymerizable small molecules within the battery system under thermal activation, electrochemical, or ultraviolet (UV) irradiation conditions, initiated by suitable initiators to form polymer electrolytes [84,85]. For MPCEs, the MOF particles are uniformly dispersed with monomers in the precursor solution before polymerization. This strategy eliminates complex electrolyte preparation procedures and reduces manufacturing costs, making it an effective approach to achieving a compatible electrode–electrolyte interface [86,87]. During the polymerization process, the liquid monomers can sufficiently infiltrate the pores of the cathode particles and conductive additives, then solidify into a continuous film that establishes molecular-level contact with the electrode surface after polymerization, thereby significantly reducing the interfacial resistance and facilitating Li⁺ transport. These improvements are crucial for inhibiting polarization and enhancing the rate performance of SSBs. In addition, compared with mechanically mixed CPEs, the in situ polymerized systems prevent phase separation among the polymer, salt, and filler, forming a more continuous ion-transport network. Furthermore, as monomer molecules polymerize into solid macromolecules, the highest occupied molecular orbital (HOMO) energy decreases to enhance oxidative stability. Therefore, the CPE possesses a wider electrochemical stability window to realize a higher energy density of the SSBs. At the same time, the consumption of liquid components during polymerization reduces the amount of flammable liquid, thereby improving the safety of SSBs [88,89]. Finally, a stable interfacial layer is formed on the cathode surface during the in situ polymerization process, which suppresses excessive transition-metal ion (e.g., Ni^2^⁺ and Mn^2^⁺) dissolution and contributes to the formation of a robust cathode–electrolyte interphase (CEI), ultimately prolonging the battery cycle life. In Bai’s work, the azobisisobutyronitrile (AIBN) was introduced in an ethylene carbonate/diethyl carbonate (EC/DEC, 1:1 *v*/*v*) electrolyte as a thermal initiator to generate free radicals that induce the in situ polymerization of vinylene carbonate (VC), forming a poly(vinylene carbonate)-based gel polymer electrolyte (PVCM-GPE) (Figure 7a) [18]. The PVCM-GPE exhibited a high ionic conductivity of (8.61 × 10^−4^ S cm^−1^ at 25 °C; tLi⁺ = 0.45); both the lithium-ion transference number and oxidative decomposition potential (4.8 V) were higher than those of conventional liquid electrolytes, indicating that the in situ polymerization process facilitated smoother Li⁺ transport and broadened the electrochemical stability window. Furthermore, the high-rate ionic conductivity enabled by the highly elastic PVCM-GPE with LiDFOB salt, at the same time, the stable SEIs were successfully formed at both the silicon-based anode and the NMC cathode interfaces. Inductively coupled plasma mass spectrometry (ICP-MS) analysis in Figure 7b revealed that the amount of Ni and Mn ions detected at the anode in the GPE-based system was markedly lower than that observed in conventional liquid electrolytes, suggesting that transition-metal dissolution from the NMC cathode was greatly suppressed. Additionally, 3D interfacial imaging configuration demonstrated a homogeneous, mosaic-like SEI layer uniformly covering the electrode surface (Figure 7c), indicative of a highly reversible alloying/dealloying reaction.

Practical Considerations: In situ polymerization offers excellent interfacial contact and reduced interfacial resistance, leading to improved cycle life. However, practical application requires careful management of monomer safety, potential shrinkage during polymerization, and the removal of initiator residues. Scaling up this process necessitates controlled environments and may face challenges in ensuring uniform polymerization in large-format cells.

#### 3.2.2. In Situ Growth of MOFs in Polymer Matrices

**Figure 8:**
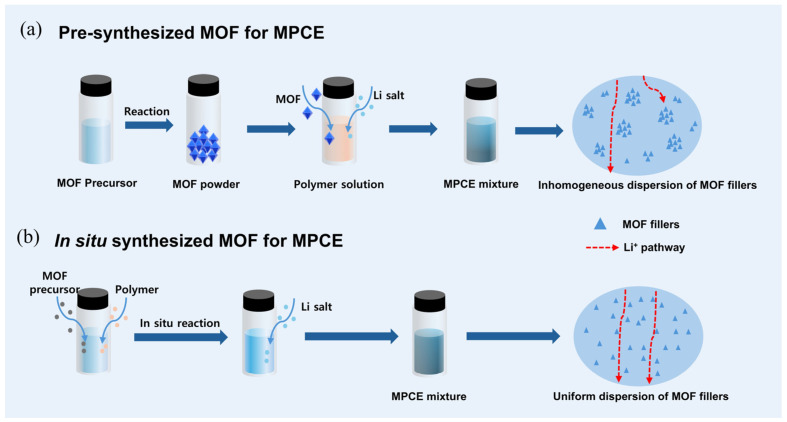
Schematic illustration of the MPCE preparation with (**a**) pre-synthesized MOF and (**b**) in situ synthesized MOF.

**Figure 9:**
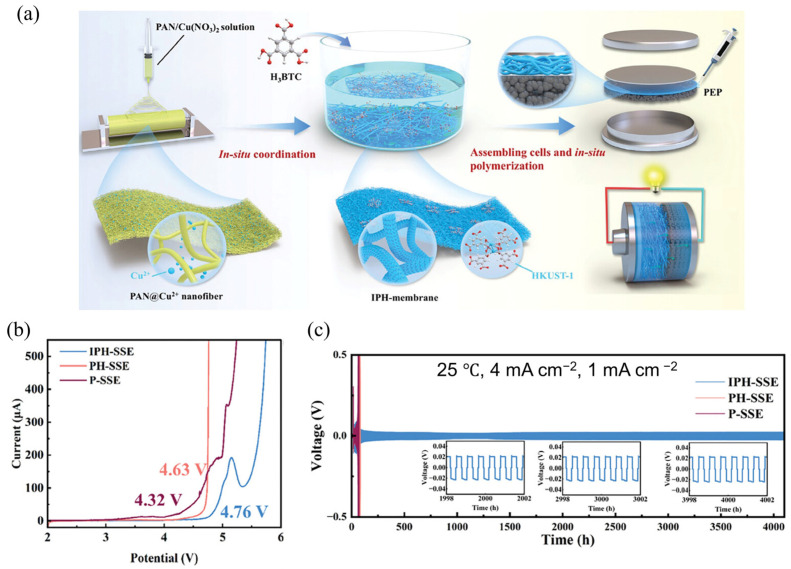
(**a**) Schematic of the electrospinning process and in situ synthesis route of IPH-SSE. (**b**) LSV curves at a scan rate of 0.1 mV s^−1^. (**c**) Li stripping–plating cycling performances of Li//Li different SSEs at 4 mA cm^−2^, 25 °C [91].

The MPCEs are typically prepared by pre-synthesizing nanoscale or microscale MOF fillers and then incorporating them into polymer matrices through a physical blending process [13]. However, the low loading and nonuniform dispersion of MOFs particles in CPEs will hinder the continuity of Li⁺ transport pathways and the stability of multiphase interfaces, as shown in Figure 8a. Therefore, in addition to optimizing the interface between the electrolyte and electrodes, improving the coupling between MOF fillers and polymers is also essential for enhancing interfacial performance. The in situ growth of MOFs within polymer matrices (Figure 8b) has been demonstrated as an effective strategy to achieve high MOF loading and homogeneous dispersion [38,70,90], thereby strengthening the interfacial compatibility between MOF fillers and polymers. For instance, Cai et al. [91] fabricated a 3D in situ coordinated PAN@HKUST-1 SSE (denoted as IPH-SSE) via electrospinning followed by chemical soaking. Subsequently, a polymer electrolyte precursor (PEP) containing 1,3-dioxolane (DOL), fluoroethylene carbonate (FEC), lithium bis(trifluoromethanesulfonyl)imide (LiTFSI), and aluminum triflate (Al(OTf)_3_) was injected into the IPH membrane and polymerized in situ, producing a blue, transparent, and flexible MPCE with excellent mechanical flexibility and intimate electrode contact (Figure 9a). The high HKUST-1 loading provided abundant cation-active sites, significantly improving the Li⁺ transference number (tLi⁺ = 0.77) and rate capability, while effectively encapsulating PAN fibers to inhibit side reactions with the lithium-metal anode. Compared with the SSE fabricated by directly mixing HKUST-1 particles with the PAN solution during electrospinning (denoted as PH-SSE) and the pure PAN fiber membrane (P-SSE), the IPH-SSE exhibited a wider electrochemical stability window of up to 4.76 V (Figure 9b), indicating enhanced oxidative stability and superior compatibility with high-voltage SLMBs. Moreover, in Li||SSE||NCM811 full-cell tests, IPH-SSE maintained stable operation up to 4.7 V, whereas PH-SSE and P-SSE underwent significant decomposition at 4.6 V and 4.3 V, respectively. And the Li symmetric cell with IPHSSE exhibited excellent stability over 4000 h for the stripping/plating cycling at 4 mA cm^−2^, 25 °C, with no significant increase in polarization (Figure 9c). Uniform and smooth surface of cycled Li anode further indicates homogeneous Li deposition realized by the in situ MOF growth within the polymer matrix, which benefited from improved interfacial compatibility and electrochemical stability of MPCEs toward active and catalytically high-voltage cathode materials.

Practical Considerations: This strategy achieves high MOF loading and excellent dispersion, enhancing mechanical properties and ion transport. The main drawbacks are the complexity of the synthesis, which often involves multiple steps and solvents, and the potential risk of damaging the polymer matrix during MOF crystallization. Scalability depends on developing simpler, more robust synthetic protocols and reducing the cost of MOF precursors.

#### 3.2.3. Gel-like Ionic Conductor

**Figure 10:**
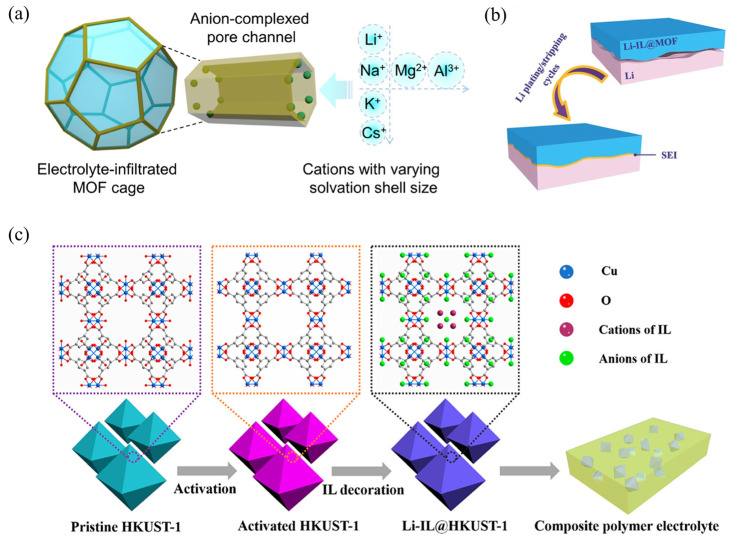
(**a**) Liquid electrolytes-infiltrated MOF cage: the anions are restricted within the MOF pores, while the MOF channels promote fast cation transport [93]. (**b**) The formed enhanced SEI layer after Li-plating/stripping cycles [94]. (**c**) Schematic representations of lithium-containing ionic liquid (Li-IL) laden gel-like ionic conductor [20].

Introducing liquid solvents, mainly including Li-containing liquid-state electrolytes (IEs) (e.g., LiClO_4_ in PC, LiPF_6_ in EC/DEC, LiTFSI in DOL/DME, et al.) and ionic liquids (ILs) (e.g., [EMIM][TFSI], [BMIm[BF], [Pyr_14_][TFSI], et al.) as plasticizers into MPCEs to form gel-like ionic conductors, called quasi-solid-state electrolytes (QSSEs), is a widely adopted strategy to reduce the contact resistance via the good interfacial wettability between the gel electrolyte and electrodes [69,92,93], thereby improving interfacial compatibility and stability. When organic plasticizers are introduced into the polymer matrix, a gel-like ionic conductor is formed when they intercalate between polymer chains to weaken intermolecular forces, disrupt crystallinity. Generally, the gel-like ionic conductor can facilitate Li⁺ transport due to the enhanced polymer chain mobility, thereby improving overall ionic conductivity. In addition, the liquid components can fill the microvoids within the electrolyte, mitigating the adverse effects of structural gaps and promoting interfacial continuity and ion-transport connectivity. However, gel ionic conductors with excessive plasticizer content usually lead to significant losses in mechanical robustness, thermal stability, and dimensional integrity, thus compromising the solid-like characteristics of the electrolyte. Therefore, achieving an optimal balance between ionic conductivity and structural stability remains a key challenge in the development of gel polymer-based electrolytes. In this regard, MOFs provide a feasible strategy to rebalance this trade-off. The organic–inorganic hybrid nature of MOFs ensures excellent interfacial compatibility with organic liquid components. The high specific surface area and open porous architecture of MOF materials provide abundant contact sites for organic liquid molecules, enabling intimate molecular-level interactions between the liquid and solid phases. This results in a pseudo-phase integration of the two components, effectively bridging the solid and liquid domains with good interfacial wettability (Figure 10a) [93]. Such a configuration guarantees continuous ion-transport pathways and rapid interfacial kinetics, even under conditions of extremely low solvent content. In addition, a uniform lithium deposition induced by this kind of interfacial wettability contributes to forming a stable SEI layer during Li stripping-plating process (Figure 10b) [94]. Wang et al. [20] reported a typical QSSE by infiltrating ILs within MOF pores then dispersing in polymer matrix, as shown in Figure 10c, in this composite electrolyte, MOFs not only act as physical hosts that selectively restrict liquid plasticizers at the molecular level through their tunable pore structures, preventing undesired flow or volatilization, but also function as inorganic fillers that reduce polymer crystallinity, introduce additional ion-conduction sites (such as –O, –F, and –N coordination groups), and reinforce the mechanical framework of the electrolyte. Consequently, MOF-integrated polymer electrolytes containing organic plasticizers can effectively balance interfacial compatibility and mechanical robustness. It should be noted, however, that introducing an excessive amount of solvent to form a gel-like conductor can reintroduce safety concerns such as liquid leakage or thermal runaway, particularly under extreme operating conditions. Hence, precise control over the MOF-to-plasticizer ratio is essential to maintain a stable quasi-solid-state configuration.

**Figure 11:**
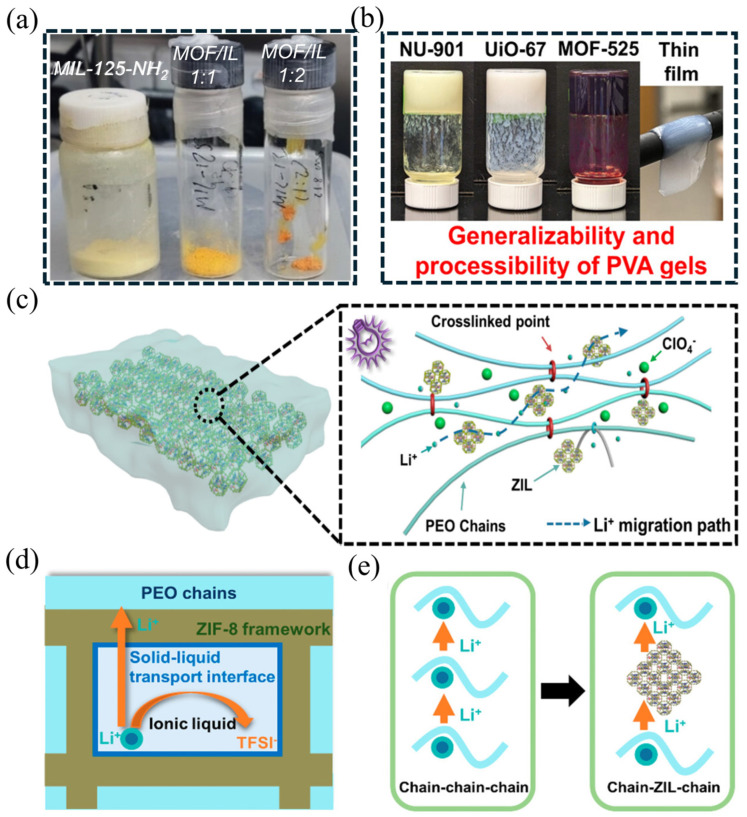
The digital photography of (**a**) gel-like ionic conductor by mixing MOF with different ratio of IL [95], and (**b**) PVA-MOF composite gels [96]. (**c**) Schematic illustration of the cross-linked composite solid electrolyte possesses both an efficient ion-conducting network and Li^+^ migration pathway. (**d**) Li^+^ transport mechanism on a solid–liquid transport interface excellent interfacial compatibility. (**e**) The new-model Li^+^ transport mechanism from “chain–chain–chain” to “chain-ZIL-chain” [97].

Salado et al. [95] used MIL-125-NH_2_ to adsorb [EMIm][TFSI] IL directly into the MOF framework at 100 °C for 12 h; the yellow-colored gel-like MOF ionic conductor was obtained when the MOF: IL weight ratio is 1:2, as shown in Figure 11a. The highly concentrated IL within MIL-125 created a better adhesion to the Li anode, thereby realizing low interfacial resistance between the electrodes and the electrolyte. Experimental results revealed that the MIL-125-IL electrolyte demonstrated an excellent ionic conductivity of 2.13 × 10^−3^ S cm^−1^ at room temperature, a high Li⁺ transference number (tLi⁺ = 0.58), and effectively suppressed lithium dendrite growth. When the MOF fillers are dispersed into a polymer such as poly(vinyl alcohol) (PVA) matrix, they can generate a free-standing polymer-MOF composite gel membrane exhibiting good elasticity and conformality (as shown in Figure 11b) [96], which possesses potential for enhancing interface contact when used as the SSEs. Xia et al. investigated the mechanism of ion transport kinetics and interfacial compatibility enhancement via gel-like electrolyte strategy [97]. In this study, a new Li^+^ transport mechanism from “chain−chain−chain” to “chain-ZIL-chain” was proposed within the solid−liquid transport interface. The ZIF-IL ionic conductor was first mixed with PEO-lithium salt matrix, then cross-linked composite solid electrolyte membranes (name as C−CSE) were constructed by in situ polymerization under ultraviolet irradiation. ZIF-8 MOF nanoparticles on PAN fibers (Figure 11c). The research demonstrated that the open porous structure of MOFs simultaneously offers numerous atomic-level contact sites with both the electrode materials and polymer matrix. In the presence of ionic liquids, this configuration forms abundant nanoscale wetted interfaces between the electrode/CPE and MOF/polymer interfaces, thereby enhancing interfacial compatibility with reduced internal resistance (Figure 11d). based on the new-model Li^+^ transportation from “chain−chain−chain” to “chain-ZIL-chain” (Figure 11e), the resulting C−CSE exhibited a high ionic conductivity of 4.26 × 10^−4^ S cm^−1^ at 30 °C and a tLi⁺ of 0.67. In addition, this C−CSE electrolyte enabled stable operation with LiFePO_4_ (LFP) cathode, capacity retention of 76.8% beyond 120 cycles at 0.1 C. Therefore, benefiting from the efficient 3D ion-conduction network and superior interfacial compatibility induced by the MOF framework liquid plasticizer, the rapid Li⁺ transport and enhanced Li⁺ transport kinetics of MPCEs were significantly enhanced for the high-performance SSBs.

Gel-like ionic conductor strategy is also widely employed for other hybrid solid electrolytes (HSEs) without MOFs to improve the interfacial environment. Liu et al. [98] reported that the ionic conductivity of CPEs is hindered by the interphase chemical structure formed between the organic (Li_6_PS_5_Cl) and inorganic (PEO) phases, and proposed introducing ILs to “activate” the interface and facilitate Li⁺ diffusion. Magic-angle spinning (MAS) ^6,7^Li solid-state NMR analyses of the interphase structure revealed that interfacial reactions occurred between Li_6_PS_5_Cl and PEO. These reactions create an inert environment that lacks free oxygen species, which is known to mediate Li⁺ diffusion in polymer electrolytes. Thus, the short of free oxygen species results in poor Li⁺ conductivity. Because there are no strong ionic bonds formed between the cationic and anionic components, ILs exhibit low solvation energies and remain dissociated. In Liu’s work, the addition of EMIM-TFSI and PP13-TFSI was found to induce markedly different bulk and interfacial structures in the PEO matrix. Because imidazolium-based ILs exhibit low viscosity and high miscibility within PEO, effectively enhancing its ionic conductivity. ^1^H and ^13^C NMR, together with differential scanning calorimetry (DSC) analysis confirmed that EMIM prefers to reside within PEO to reduce the crystalline fraction, whereas PP13 mainly locates at the Li_6_PS_5_Cl surface. Combined with the poor miscibility of PP13 in PEO, it suggests that PP13 mainly accumulates at the PEO/Li_6_PS_5_Cl interface, serving as both a wetting agent and a Li⁺ transport bridge, exhibiting the symmetric cell a more stable overpotential.

Practical Considerations: Gel-like conductors significantly improve wettability and ionic conductivity, often reaching values comparable to liquid electrolytes. The primary trade-off is a reduction in mechanical strength and thermal stability, which can compromise safety and the “solid-state” nature of the battery. For scale-up, the long-term stability of the plasticizer within the MOF/polymer matrix and the associated safety risks under abuse conditions must be thoroughly evaluated.

#### 3.2.4. Composite Cathodes (CC) Design

**Figure 12:**
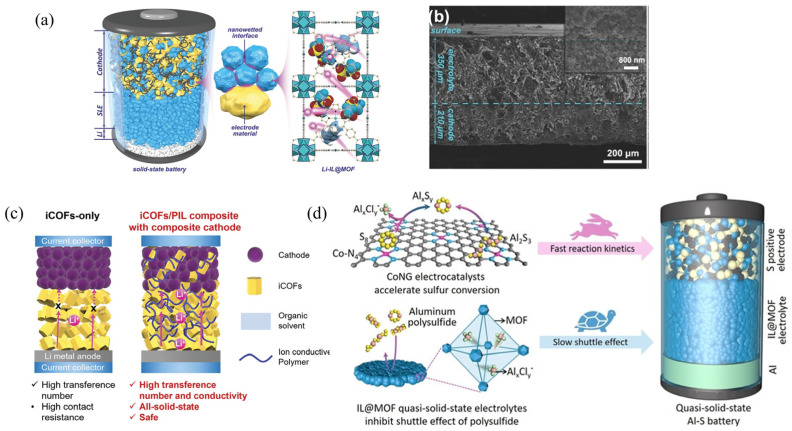
(**a**) Schematic illustration for the nanowetted interfacial mechanism of the Li-IL-MOF in the composite cathode. (**b**) SEM image of the cross-section of the composite LFP cathode [94]. (**c**) Comparison of Li^+^ transport in pure iCOF-based electrolytes and iCOF/PIL CPE with composite cathode [108]. (**d**) The working mechanism of IL@MOF conductor and CoNG electrocatalyst within the quasi-solid-state battery [109].

Incorporating solid ionic conductors as the catholytes into the active material to form composite cathodes (CC) is an effective strategy to further reduce the interfacial resistance between the SSE and the cathode [99,100]. The CC is typically prepared by mixing the active cathode material, catholyte, and electronic conductive additive. The catholyte works both as an ionic conductor and mechanical binder, facilitating efficient ion transport and enhancing interfacial electrochemical reactions. Therefore, these designed CC cooperate with the CPEs within SSBs can achieve high ionic conductivity, promote Li^+^ transport, and minimize contact resistance [99,101]. For the common CPEs, most design strategy for CC involves using semicrystalline polymer electrolytes without inorganic ion conductors as the cathode electrolyte, even when the SSE is a polymer–ceramic composite. Because comparing to inorganic materials, these polymers possess superior flexibility and plasticity [102,103,104], allowing them to form continuous adhesive networks between particles, which promotes the formation of a flexible interfacial layer between the cathode material (e.g., LFP or NCM) and the bulk SSE, thereby improving ion transport and interfacial compatibility. Hu et al. [105] fabricated a PI–LLZTO/PVDF CPE by coating a mixture of LLZTO/PVDF onto a porous polyimide (PI) membrane, then employed the composite polymer of succinonitrile (SN)/PVDF as the catholyte and binder for the CC preparation. The optimal cathode composition was obtained with 81.1 wt% NCM, 4 wt% PVDF, 6.75 wt% Super-P, 6.75 wt% SN, and 1.4 wt% LiTFSI, significantly enhancing ion transport and reducing interfacial resistance. The solid-state pouch cell exhibited excellent cycling stability at room temperature, maintaining 94.9% of its initial capacity after 80 cycles at 0.1 C and demonstrating reliable safety performance. However, MOFs are often used for the CC design, due to their 3D open solid framework, achieving a nanowetted interface. Wang et al. [94] reported a composite LFP cathode consisting of 41.7 wt% LFP, 16.6 wt% active material, and 41.7 wt% Li-IL@MOF ionic conductor (Figure 12a). In the cathode layer, LFP grains are uniformly encapsulated by conductive carbon and Li-IL@MOF nanocrystals, forming a compact three-dimensional conductive network. This architecture endows the composite with excellent electronic and ionic conductivity. SEM images in Figure 12b clearly reveal that the composite cathode is seamlessly laminated with the solid electrolyte layer, without noticeable interfacial voids, which facilitates efficient ion transport across the interface and markedly reduces interfacial resistance. Consequently, even with a thick CC layer of up to 350 μm, the total resistance of the thick-electrode LFP solid-state battery before cycling remains as low as ~410 Ω. These results confirm the unique interfacial wetting mechanism induced by the Li-IL@MOF ionic conductor, which effectively enhances interfacial contact within the composite electrode. Extending to other framework materials, for instance, the pure ionic covalent organic framework (iCOF)-based SSE exhibited high interfacial resistance due to the presence of large voids between iCOF particles, leading to poor ionic conductivity [106,107]. To address this problem, Huang’s group applied a poly(ionic liquid) (p(BVIm-TFSI), named as PIL) to fill the iCOFs (TpPa-SO_3_Li and DMTHA-Si-Li) voids, fabricating iCOF/PIL CPEs [108]. At the same time, the CC was developed to replace the conventional cathode material by introducing iCOF/PIL CPE into the cathode component, which was prepared with 70 wt% active material (LFP), 10 wt% CPE, 10 wt% PVDF, and 10 wt% carbon black. Notably, the fabricated iCOF/PIL CPE in their work attributed the highest solvent- and plasticizer-free COF-based ionic conductivity (>1.30 × 10^−3^ S cm^−1^) (Figure 12c). And the Li//iCOF/PIL CPE//CC SSB exhibited stable performance at higher rates of 1 C and extended lifetime of 800 cycles with >80% capacity retention. Furthermore, incorporating IL@MOF into the sulfur-based cathode effectively reduces interfacial resistance, enabling rapid ion transport and significantly suppressing the polysulfide shuttle effect, thereby stabilizing the reversible sulfur conversion process. Meanwhile, the introduction of catalytically active cobalt/nitrogen co-doped graphene (CoNG) nanosheets as the sulfur host further accelerates the sulfur redox kinetics (Figure 12d) [109]. As a result, the quasi-solid-state Al–S battery assembled with the S@CoNG-IL@MOF composite cathode and solid electrolyte successfully achieves both high energy density and prolonged cycling stability.

Practical Considerations: The composite cathode design is highly effective for minimizing interfacial resistance within the cathode layer itself, enabling the use of thick electrodes for high energy density. The challenges lie in the complex formulation process, potential trade-offs between ionic and electronic conductivity, and the added manufacturing complexity of integrating the solid ionic conductor (catholyte) homogeneously with active materials. Scalable coating techniques must be adapted to handle these composite slurries.

## 4. Current Challenges and Limitations

Despite the remarkable progress achieved in the development of MOF–polymer composite electrolytes, several critical challenges continue to impede their large-scale practical implementation. First, the molecular-level interaction mechanisms between MOF fillers and polymer matrices remain insufficiently understood. It is still unclear how variations in metal centers, organic linkers, and pore architectures influence polymer chain dynamics and subsequently regulate ion migration pathways. The lack of systematic mechanistic insights makes it difficult to perform predictive materials design. In addition, the correlations between MOF structural characteristics and their electrochemical performance are not yet well established. Key parameters such as pore size, specific surface area, pore polarity, and metal–oxygen bond strength directly affect ionic conductivity, but their quantitative influence requires further investigation. Reproducibility also poses a substantial challenge. The performance of MPCEs is highly sensitive to synthesis routes, the dispersion quality of MOFs, and processing conditions such as temperature, solvent environment, and pressure. Even minor variations can lead to pronounced fluctuations in electrochemical performance, undermining research reliability and complicating scale-up. Finally, the high fabrication cost of MOF material—including expenses associated with precursors, energy consumption, and complex purification processes—remains a major obstacle to commercial deployment. Collectively, these challenges constrain the economic feasibility of MPCEs and hinder their potential for industrial-scale application.

## 5. Conclusions and Perspectives

In summary, MPCEs represent a promising direction for the development of SSEs, which integrate the flexibility of polymers with the structural functionality of MOFs. This review summarizes the fabrication methods of MPCEs and then investigates the functional role and mechanisms of MOF fillers in enhancing composite electrolyte performance, particularly their remarkable potential in accelerating ion transport, suppressing dendrite growth, and improving electrochemical stability. Subsequently, strategies for interfacial engineering of MPCEs are discussed, including in situ polymerization and in situ synthesis of MOF fillers to optimize the electrolyte architecture, gel-like conductor strategy through ladening liquid plasticizers to improve interfacial compatibility, and the design of composite cathodes to reduce interfacial resistance while enhancing interfacial stability and compatibility. In light of the current challenges and limitations, advancing MPCE materials from laboratory research to practical application will require several strategic research directions. First, future studies should leverage in situ/quasi-in situ characterization techniques in combination with multiscale simulations (e.g., DFT and MD) to further elucidate the molecular-level interactions between MOFs and polymer matrices. Such approaches will help clarify how metal centers, linker polarity, and pore architectures regulate polymer chain dynamics, thereby enabling predictive materials design. Second, it is essential to establish quantitative correlations between MOF structural parameters, such as pore size, functional group polarity, and metal–oxygen bond strength, and the resulting electrochemical performance. Developing a unified structure–performance evaluation framework will also facilitate meaningful comparisons across different studies. Finally, efforts should focus on improving the reproducibility of both materials and fabrication processes, developing scalable and controllable synthesis routes, and reducing production costs through optimized reaction pathways and cost-effective precursors, ultimately enhancing the economic feasibility of MPCEs. With continued research growth, further breakthroughs are expected in the near future.

## Figures and Tables

**Table 2:** Summarizes the characteristics of various interface engineering methods.

Strategy	Mechanism	Applicability/Complexity	Strengths	Limitations	Typical σ/tLi⁺	Ref.
In situ polymerization	Monomer infiltration, then polymerization, forming an intimate interphase	Medium–high; requires monomer compatibility	Low interfacial resistance; conformal contact	Shrinkage; limited to monomer-compatible systems	σ ~10^−4^–10^−3^ S/cm; tLi⁺ ~0.4–0.6	[12]
In situ MOF growth	MOF grown inside the polymer matrix for uniform dispersion	High; synthesis-sensitive	High MOF loading; strong MOF–polymer coupling	Polymer damage risk; synthetic control needed	σ ~10^−4^–10^−3^ S/cm; tLi⁺ up to ~0.77	[70]
Gel-like ionic conductor	Liquid/plasticizer inside MOF pores improves wettability	Low–medium	High σ; excellent interface wetting	Mechanical weakness; safety concerns	σ up to ~10^−3^ S/cm	[71]
Composite cathode design	Catholyte integrated into the cathode, reducing interfacial resistance	Moderate	Reduced voids; suitable for thick electrodes	Complex formulation; mass tradeoffs	Low R_interface; σ depends on the system	[72]

## Data Availability

No new data were created or analyzed in this study.
